# Fatigue in Multiple Sclerosis: Misconceptions and Future Research Directions

**DOI:** 10.3389/fneur.2016.00122

**Published:** 2016-08-02

**Authors:** Thorsten Rudroff, John H. Kindred, Nathaniel B. Ketelhut

**Affiliations:** ^1^Department of Health and Exercise Science, Colorado State University, Fort Collins, CO, USA

**Keywords:** multiple sclerosis, perceptions, performance fatigability, neuroimaging, questionnaires

## Abstract

Fatigue is one of the most disabling side effects in people with multiple sclerosis. While this fact is well known, there has been a remarkable lack of progress in determining the pathophysiological mechanisms behind fatigue and the establishment of effective treatments. The main barrier has been the lack of a unified definition of fatigue that can be objectively tested with validated experimental models. In this “perspective article” we propose the use of the following model and definition of fatigue: *the decrease in physical and/or mental performance that results from changes in central, psychological, and/or peripheral factors*. These changes depend on the task being performed, the environmental conditions it is performed in, and the physical and mental capacity of the individual. Our definition and model of fatigue outlines specific causes of fatigue and how it affects task performance. We also outline the strengths and weaknesses of commonly used measures of fatigue and suggest, based on our model and definition, new research strategies, which should include multiple measures. These studies should be mechanistic with validated experimental models to determine changes in central, psychological, and/or peripheral factors that explain fatigue. The proposed new research strategies may lead to the identification of the origins of MS related fatigue and the development of new, more effective treatments.

Fatigue is the most common and disabling symptom experience by people with multiple sclerosis (PwMS). Up to 92% of PwMS are affected by fatigue, which strongly influences quality of life (1). However, fatigue remains poorly understood and PwMS continue to suffer from a lack of effective fatigue treatments. Despite significant effort to elucidate the pathogenic mechanisms of fatigue, current knowledge is limited. Several factors contribute to the lack of progress in fatigue research, but the most important factor is that “fatigue” is often not clearly defined or is used without meaningful measurements in clinical and research settings (2). Kluger et al. (3) states: “Current treatments are non-specifically targeted to a vaguely defined symptom with unsatisfactory outcomes.” Providing further support to these statements, a recent Cochrane Review (4) on exercise therapy for fatigue in MS concluded there are important methodological issues to overcome. Heine et al. (4) reported most studies did not: explicitly include PwMS who experienced fatigue, use a validated measure of fatigue as the primary outcome, or target fatigue specifically. Berger (5) questions whether MS related fatigue can be treated and improved with current disease-modifying drugs, e.g., amantadine, methylphenidate, and modafinil, without having a precise definition of fatigue.

In this “perspective paper” we propose a model of fatigue designed to give clinicians and researchers a better understanding of fatigue, critically review current fatigue measures used in MS, and provide suggestions for new research strategies to better understand fatigue in MS.

## Definitions of Fatigue

Many studies investigating fatigue have failed to objectively define fatigue, and those that did, have used varying definitions. Furthermore, the origins of fatigue vary between conditions and research in some diseases, such as MS, has failed to fully understand the difference between fatigue and related phenomena, such as depressed mood or sleep disorders (3). As a result, Kluger and colleagues (3) recently proposed a unified taxonomy for fatigue in neurological disorders that classified fatigue into two major domains: performance fatigability and perceptions of fatigue. Performance fatigability was defined as the magnitude or rate of change in a performance criterion relative to a reference value over a given time of task performance. Perceptions of fatigue was defined as a subjective sensation of weariness, increasing sense of effort, mismatch between effort expended and actual performance, or exhaustion.

**Figure 1:**
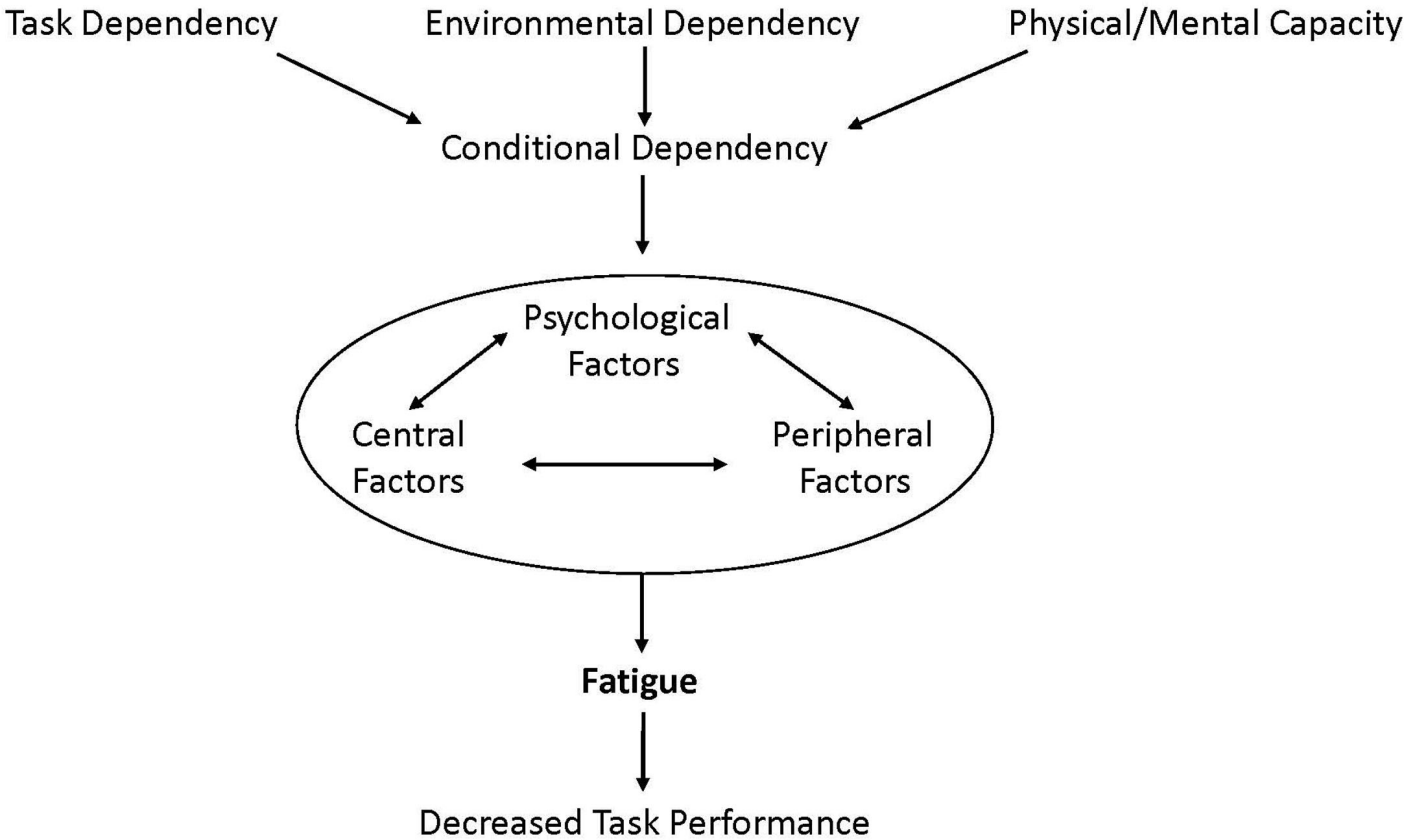
**Fatigue is defined as the decrease in physical and/or mental performance that results from changes in central, psychological, and/or peripheral factors**. These depend on the task being performed, the environmental conditions it is performed in, and the physical and mental capacity of the individual (conditional dependency). Importantly, fatigue is greatly affected by the factors of conditional dependency and the interactive changes in central, psychological, and/or peripheral factors that cause fatigue.

We propose fatigue should be defined as: the decrease in physical and/or mental performance that results from changes in central, psychological, and/or peripheral factors. Indeed these factors all have *conditional dependency* in that the changes in central, psychological, and peripheral factors of fatigue depend on the task being performed, the environmental conditions it is performed in, and the physical and mental capacity of the individual (Figure 1). Central factors of fatigue are related to changes within the function of the central nervous system, such as neurotransmitter levels and intrinsic neuronal excitability, while psychological factors of fatigue include mood disorders, perceptions of effort, motivation, temporal and performance feedback, and arousal. Finally, peripheral factors of fatigue refer to physiological changes, such as pH, muscle contractility and excitability, and substrate availability. Importantly, the phrase *factors of fatigue* is used because there are many changes from a variety of locations that can all interact to contribute to fatigue. As a result, terminology that refers to a specific location of the fatigue, such as central, peripheral, and muscle, should be avoided as fatigue is almost never focused to a specific location.

Task dependency has been identified and discussed as a component of fatigue in healthy individuals for several decades (6–9). A variety of human studies indicate that fatigue is not caused exclusively by any common set of factors, but depends on the type of cognitive or motor task that is being performed. However, many conditions beyond the specific task being performed can affect fatigue. One distinct difference between our model and previous models (3, 10) is the concept of conditional dependency, which considers not only task dependency but also how the condition it is performed in and the physical and mental capacity of the individual affect fatigue. Environmental dependency considers how factors of the environment affect fatigue, such as temperature and comfort level of the individual in the space. This can be of particular importance in MS, as warm environments can greatly affect physical abilities of PwMS (11). Finally, fatigue depends on a person’s physical and mental capacity, which includes the consideration of differences in fatigue before and after the onset of a disease or day-to-day variation in disease status due to disease progression or temporary relapse. These different conditions each interact with each other, as well as the central, psychological, and peripheral factors of fatigue discussed above. Our model not only displays how the varying conditions affect the interactive/factors but also the importance of considering the impact of factors, such as depression, general lack of motivation, and altered states of arousal, when measuring fatigue. This is most easily seen in the reported interactions between depression and fatigue in PwMS, where studies have shown that reported fatigue levels often decrease once depression is accounted for (12, 13). Therefore, what is being reported as “fatigue” is often a manifestation of an underlying condition, which may or may not be related to an individual’s ability to perform a task.

## Assessment of Fatigue in MS

The two interactive subtypes (perception and performance) of fatigue proposed by Kluger and colleagues (3) are each important when considering the impact of fatigue on PwMS. Increased perceptions of fatigue can have a significant impact on activities of daily living, mood, and likelihood to engage in social activities, resulting in a reduced quality of life. While performance fatigability may only impact PwMS during a task, it has meaningful impact on an individual’s ability to perform activities of daily living and to live independently. Furthermore, performance fatigability impacts an individual’s exercise capacity, which is important in managing the symptoms of MS (14).

### Subjective Measurements of Fatigue

A remarkable number of questionnaires have been developed to assess fatigue. They range in length from single-item scales [e.g., visual analog scale (VAS)], to multidimensional scales claiming to assess various dimensions of fatigue, such as physical vs. mental [e.g., modified fatigue impact scale (MFIS)]. Most of the items on these assessments are similar and correlate very well with each other. One major issue with fatigue questionnaires is the construct contamination that mars the validity and specificity of such scales (2). For example, fatigue questionnaires often include questions about tiredness and cognition, which are not always associated with fatigue (2).

Self-report questionnaires can be influenced by other symptoms of MS, require patients to make difficult reflective assessments, and are completely subjective. Despite these limitations, the fatigue severity scale (FSS) (15) and the MFIS (16) are commonly *the only measures* of fatigue in many studies [e.g., Ref. (17–19)]. Most of these studies were incapable of demonstrating responsiveness to changes over time to therapeutic interventions, likely because of a lack of specificity in the questions or underlying factors of MS that may be reported as fatigue. However, questionnaires will continue to have value, especially for measuring perceptions of fatigue, until quick and easy objective clinical assessments are available.

### Objective Measurements of Fatigue

Objective measures of fatigue are limited to variables obtained *during* physical or mental tasks, and measured *pre-, during-*, and *post-task*. The measures of these tasks can be objectively quantified in research and clinical settings. Fatigue during motor tasks is usually characterized by the decline in peak force, power, accuracy, or speed from pre- to post-task. During cognitive tasks, fatigue is often measured as declines in reaction time or accuracy-over-time on continuous tasks. While several studies have attempted to objectively quantify MS related factors of fatigue using the neuroimaging techniques (described below), they have failed to measure factors of performance fatigability. Separating fatigue into the motor and cognitive tasks or domains provides objective assessments that are less likely to be contaminated by other symptoms of MS and help distinguish fatigue from related factors, such as reduced cognitive processing speed, sleep disorders, depressive symptoms, and anxiety (20, 21). Therefore, it is suggested that measures, such as sleepiness and mood be included as covariates, when investigating MS related fatigue. Depression, mood, anxiety, cognitive–behavioral factors, motivation, sleep disorders, and low sense of control contribute to fatigue (22–24). How people then react to these underlying conditions may serve to prolong or worsen fatigue. Specifically, depression affects a significant proportion of PwMS during their life span (25). Accordingly, Bakshi et al. (26) found that depression is associated with MS related fatigue, indicating that depression should be controlled for.

Once fatigue is defined, valid tasks and indices may be employed to measure the contributing factors of fatigue. Several technologies are available to investigate the changes in these factors during tasks, including: electromyography, metabolic measures, transcranial magnetic stimulation, magnetic resonance imaging (MRI), near infrared spectroscopy, and positron emission tomography (PET). Since MS related fatigue is a multifactorial problem, it is important to use multiple instruments to investigate fatigue, and identify the central, psychological, and peripheral factors contributing to the decrease in task performance.

## Factors Contributing to Fatigue in MS

### Central Factors

Central factors contributing to fatigue include neurotransmitter levels, inflammation, neuronal excitability, substrate utilization/transport, axonal conduction velocity, and many others. CNS inflammation is a hallmark of MS and has been suggested to play a role in MS related fatigue, although the current literature reports conflicting findings (27). For example, some researchers have shown associations between cytokine levels and fatigue questionnaire scores (28–30), while others have shown no association between cytokine or c-reactive protein levels with fatigue questionnaires (31, 32). Therefore, future research is needed to determine whether CNS inflammation, in addition to many other factors, is a potential central factor of fatigue in MS.

Neuroimaging studies have started to provide direct evidence of the central factors of fatigue in MS. Roelcke et al. (33) used ^18^F-fluorodeoxyglucose-PET to measure cerebral glucose metabolism in PwMS. They found significant hypometabolism throughout the brain in fatigued PwMS, based on FSS scores, suggesting dysfunctional cerebral activity might be responsible in MS related fatigue. However, it cannot be ruled out that cerebral hypometabolism is caused by other symptoms, such as depression, which were not specified in the fatigue questionnaire.

Functional-magnetic resonance imaging (fMRI) is a prominent neuroimaging technique used to investigate cerebral activity. Although this technique provides the opportunity to detect brain regions involved with motor or cognitive tasks, the interpretations and conclusions resulting from fMRI studies are often misleading. A good example is a recent fMRI study that examined cognitive fatigue in PwMS (34). A cognitive task was performed within the MRI scanner and the VAS was used during the task to measure “state” cognitive fatigue (35). The authors concluded that PwMS had increased brain activity in the caudate, compared to healthy controls, resulting in greater VAS scores. However, there was no significant group x time interaction, indicating the task elicited the same change in task performance (fatigue) in both the MS and healthy groups. Furthermore, performances on the neurophysiological tests were not different between groups. Similar findings were seen in Rocca et al. (36), where fMRI during a motor task was obtained from PwMS with and without “fatigue” and matched healthy controls. Brain activation strategies were different between the groups during the motor tasks without differences or changes in task performance. Task performance was similar between the investigated groups in both studies, which suggests that the findings only help explain underlying factors that contributed to the initial difference in fatigue between the groups (perceptions of fatigue), not performance fatigability. If altered brain activation strategies lead to decreased task performance, then conclusions about performance fatigability could have been made.

It has been suggested that an imbalance of dopamine in the CNS and immune system plays an important role in fatigue (37). Neuroimaging studies have shown that brain regions with impaired structure and function are heavily innervated by dopaminergic neurons (38, 39). Rönnbäck and Hansson (40) stated that “mental fatigue” is also associated with impaired glutamate neurotransmission and hypothesized that there might be a genetic failure preventing astroglial glutamate transporters from upregulating. In our model, the aforementioned findings refer to perceptions which are modulated by central and psychological factors, and if they do not change during the performance of a task they should only be classified as a factor of perceptions of fatigue.

Some studies showed associations between fatigue and lesion load/location and the degree of gray matter atrophy (41, 42). They found that MS related fatigue, assessed by the MFIS, FSS, and MRI, is at least partially associated with disruption of frontal and parietal pathways and cortical areas involved in cognitive/attentional processing. However, based on our proposed model of fatigue, it remains unknown whether structural brain changes, such as atrophy and lesion load, are factors of performance fatigability since they have not been associated with an objective measure of performance fatigue.

Together, these studies show that central factors of fatigue undoubtedly contribute to perceptions of fatigue in PwMS. However, the necessary measures of performance fatigability have not been performed in these studies, and therefore it is still unknown whether these central factors contribute to performance fatigability in PwMS.

### Psychological Factors

Psychological factors, such as perceived effort, subjective sense of worsening performance over time, motivation, and cognitive impairment are contributors to fatigue (43). Serotonin (44) and dopamine (45) are just two examples of central factors that influence psychological factors and play an important role in fatigue. Engström et al. (46) showed that PwMS who have high fatigue demonstrate reduced mesocorticolimbic connectivity compared to healthy adults during a complex working memory task. Finke et al. (47) showed that high fatigue scores in PwMS were negatively correlated with resting-state mesocorticolimbic connectivity. These findings suggest that MS related fatigue is associated with reduced connectivity between the regions innervated with dopamine, possibly due to reduced dopamine levels.

### Peripheral Factors

Fatigue in PwMS can also arise from one or several of the peripheral factors that were described above. Slowing of muscle contractile properties (48–51), decreased muscle oxidative capacity (48, 52), impaired excitation–contraction coupling (50, 53), and altered muscle metabolic response to exercise (50, 53, 54) may contribute to fatigue in MS. Sharma et al. (50) showed that intramuscular components contribute to fatigue in MS by demonstrating that greater decreases in phosphocreatine and intracellular pH was associated with greater force reduction (performance fatigability). In this context, it is important to mention muscle afferent feedback, which includes the possibility that metabolites can alter CNS motor output (55).

## Final Thoughts and Future Research Directions

When compared to advances made in other domains of disease status and disability in PwMS, fatigue continues to lag behind. The lack of progress is largely due to the varying subjectivity in the definition and assessment of fatigue between research groups. Our proposed theoretical model provides specific areas of objective fatigue assessment that can be applied in research and intervention settings. Because of the complexity of fatigue in PwMS, it is important that future studies should not only account for covariates, including depression and sleepiness, but also require integration of multiple measures directed at the different factors that influence fatigue. Currently, several techniques are available to measure many of the factors that could contribute to fatigue in PwMS. Central factors can be examined via neuroimaging techniques, such as PET and MRI, psychological factors via the Brief Repeatable Battery of Neuropsychological Tests (56), and peripheral factors with electromyography and MR spectroscopy to name a few.

In this “perspective paper” we proposed a standardized definition of fatigue and identified factors that contribute to fatigue. However, this list is not exhaustive; *our model is hypothetical* and further research is needed to elucidate all mechanisms of MS related fatigue and to validate our proposed model. Research studies should focus on clearly defined outcome variables, which *contribute to fatigue* and not primarily on the location of fatigue. Future research studies on MS related fatigue should be extended to include psychological screening to determine underlying conditions, which may or may not impair task performance but should be distinguished from fatigue. We propose fatigue should be defined as: the decrease in physical and/or mental performance that results from changes in central, psychological, and/or peripheral factors. Importantly, these changes depend on the task being performed, the environmental conditions it is performed in, and the disease status of the individual.

An example for the objective measurement of fatigue in PwMS with defined outcome variables is a study by Sharma et al. (50). Fatigability of the anterior tibialis muscle was quantified in PwMS and controls during intermittent electrical stimulation. During stimulation, the decline in tetanic force, phosphocreatine, and intracellular pH was greater in PwMS than in controls, indicating an abnormal intramuscular component of fatigue in MS. Importantly, this study eliminated the influence of perceptions of fatigue since it did not involve voluntary muscle activity.

While the study design described above was ideal to identify several peripheral factors of performance fatigability, future studies must include voluntary muscle activity, which incorporates central and psychological factors, to fully understand fatigue. This can be accomplished by measuring changes in peripheral factors (muscle strength and activity, pH, glycogen, etc.), as well as measures of central and psychological factors (dopamine, motivation, perceived effort, etc.). For example, the design used by Sharma et al. (50) could be expanded to include voluntary muscle activity, and neuroimaging techniques (fMRI and PET) could be applied to measure changes in central and psychological factors. Perceptions of fatigue should be monitored in this example using the techniques, such as the Borg scale of perceived exertion. The associations of these measures then may provide insights to the origins and mechanisms of MS related fatigue.

By using a uniformed understanding and measurement of fatigue, progress may finally be made in effectively treating the symptoms of fatigue and improving quality of life in PwMS.

## Author Contributions

TR, JK, and NK contributed to drafting the article and revising it critically for important intellectual content. All authors approved the final version of the manuscript.

## Conflict of Interest Statement

The authors declare that the research was conducted in the absence of any commercial or financial relationships that could be construed as a potential conflict of interest.
